# Developmental biology teaching - the importance of a practical approach

**DOI:** 10.12688/f1000research.6559.1

**Published:** 2015-05-26

**Authors:** John F Mulley

**Affiliations:** 1 School of Biological Sciences, Bangor University, Bangor, Wales, LL57 2UW, UK

**Keywords:** Education, teaching, developmental biology, laboratory practicals, biological sciences, active learning

## Abstract

The huge growth in knowledge in many areas of biological sciences over the past few decades has created a major dilemma for those of us in higher education, for not only must we adequately and efficiently convey these new facts and concepts to our students, we must also ensure that they understand and appreciate them.

The field of developmental biology has witnessed such a massive growth in knowledge since the mid-1980s, driven mainly by advances in cell and molecular biology, and the development of new imaging techniques and tools. Ensuring that students fully appreciate the four-dimensional nature of embryonic development and morphogenesis is a particular issue, and one that I argue can only be properly learned via direct exposure to embryos via laboratory practicals.

## Background

Developmental biology is a subject with a long and rich history, and also one in which there have been tremendous and rapid advances over the past few decades. Indeed, it has recently been suggested that these advances, especially those in genetics and genomics and cell (particularly stem cell) biology, have been so great as to require a fundamental shift in developmental biology itself ( St Johnstone, 2015). The British Society for Developmental Biology (BSDB) has twice in recent years considered renaming itself the “British Society of Developmental and Stem Cell Biology” ( Stem Cells in Developmental Biology: a debate at the BSDB) and the flagship journal *Development* made a conscious effort to “…become an important player in the stem cell field” ( Pourquié, 2012). Regardless of these apparent insecurities and image problems, the undoubted growth in knowledge regarding the embryonic development of an ever-growing number of species and the associated explosion of new facts and concepts, together with an extensive historical body of work, raises the issue of how best to convey these facts and concepts to undergraduate students. Whilst this issue is not necessarily unique to developmental biology ( Schwartzbauer, 2003; Tunnicliffe & Ueckert, 2007; Yeong, 2012), it has been argued that the absolute requirement for four-dimensional thinking to truly understand the patterns and process of embryonic development is one not necessarily shared with other disciplines in the biological sciences ( Hardin, 2008). It has also been suggested (Wood, 2008) that the issue of teaching concepts rather than facts is more complicated in developmental biology, as much of what is taught can be considered to be both a fact and a concept. Thus it is the way that we ask students to use this knowledge that determines whether they regurgitate it as a fact, or fully appreciate it and apply it as a concept. Given these considerations, how are we to ensure that students on developmental biology courses, and, indeed, general biology, zoology or biomedical courses, not only learn, but *understand* the principles of embryonic development? I would argue that this is only possible through direct exposure to embryos and developmental biology techniques in laboratory-based practicals.

It is widely acknowledged that students learn best by doing and by having the opportunity to put what they have learned into practise ( Kolb, 1984; Moon, 2013). In addition, the ability to design and carry out experiments is a fundamental requirement of training in the sciences ( Hofstein & Lunetta, 2004; Kirschner, 1992; Kirschner & Meester, 1988). However, it must be borne in mind that students by their very nature do not usually *practice* science *per se*, but rather are *learning to practice* science ( Kirschner, 1992, see also Adams, 2009 for some exceptions). Science teaching should therefore aim to familiarise students with the way that science works ( Allen & Tanner, 2003; Allen & Tanner, 2005; Hmelo-Silver, 2004; Kendler & Grove, 2004), whilst remembering that students lack the theoretical knowledge, sophistication and experience of a researcher. In the same way, purely discovery-based approaches can often fail to properly engage learners with the material, and guided discovery better promotes constructivist learning ( Mayer, 2004). In short, behavioural activity does not equal cognitive activity and we must be careful to consider *why* we are getting students to perform particular tasks.

Kirschner (1992) identified three motives for implementing practicals:

1. Service to theory – the practical is used to illustrate or affirm theories taught in another setting. In this way the practical is subservient to other forms of instruction and also subservient to theory, where in fact theory and practice are interdependent.
2. Discovery as the only way to achieve meaningful learning – the practical is used to provide discovery learning and process approaches in the absence of prior theoretical context. However, this relies on an assumption that reception learning cannot be meaningful.
3. As a means to distil insight or understanding from empirical work – the practical is used to provide experience to help students to understand a theory. This approach assumes that meaningful learning can take place (i.e. learners can make sense of their observations) in the absence of a robust conceptual framework.

Others propose that practical sessions are better suited to the development of specific skills (and to counter the shift towards the teaching of generic “key” skills); to learn the academic approach and to allow students to experience phenomena ( Abrahams & Millar, 2008; Collis *et al.*, 2008). In addition, co-operative learning and group work allows students to experience multiple roles and aids in the development of collaborative skills and, together with opportunities for group discussion and personal reflection contributes towards the experiential learning cycle ( Kolb, 1984; Kolb & Kolb, 2005). Clearly, the practical approach is a powerful one, and one that can have a great impact not only on the learning process in a particular course but more widely on a student’s whole skill set and entire undergraduate experience.

## Practical developmental biology

Towards the end of my Postgraduate Certificate in Higher Education (PGCertHE) in 2013, I designed a 20 credit practical-based 3 ^rd^ year module (‘Practical Developmental Biology’) for undergraduate students in the School of Biological Sciences at Bangor University and this ran for the first time in semester 2 of the 2014/15 academic year. The rationale behind the course (reflected in its intended learning outcomes) was to provide students with an understanding of key models and techniques used to study animal development; to enable them to develop practical skills in embryology and to give them the opportunity to combine background knowledge and independent research to interpret experimental results and solve problems. Interestingly, attempts to involve the students in the design of course content via a brief pre-module survey (“What are you hoping to get out of this module?”; “Are there any particular resources that you’d like to have available, either during a module or before it starts?”; “Is there anything that you have particularly liked in other modules that I can steal for this one?”; “Is there anything you have particularly disliked in other modules that I should avoid in this one?”) met with limited success (16% response rate), although such low response rates are common, possibly as a result of survey fatigue ( Porter *et al.*, 2004; van Mol, 2014). The module comprised 21 three-hour practical sessions, ranging from fairly basic single sessions involving direct observation of chicken ( *Gallus gallus*), zebrafish ( *Danio rerio*) and axolotl ( *Ambystoma mexicanum*) embryos and setting up crosses of *Drosophila melanogaster*, to more complex *in situ* hybridisation and immunohistochemistry experiments to detect gene expression and protein distribution which ran across multiple sessions over several weeks. In addition to developmental biology skills, students were also given the opportunity to practise key molecular biology techniques, such as DNA/RNA extraction, PCR and RT-PCR, agarose gel electrophoresis, as well as to develop and improve existing skills in general numeracy, pipetting and microscopy, among others. The module was assessed via three pieces of written work (2000–2500 word combination practical write-ups and technique reviews, each worth 25%) and a laboratory notebook (also worth 25%).

## Discussion

Embryonic development provides the link between the genes and gene frequencies learned about in genetics modules and the animals bounding around in fields which students (especially those on Zoology courses) so desire to see on field trips. The formation of the nervous and sensory systems during development dictates behaviour, as all behaviour is ultimately dependent on the ability to sense and respond to the environment and it could also be argued that the embryo is the most important level for selection to act on to change morphology. An understanding of developmental biology is therefore important for students of the biological sciences, and vital for those on Zoology degrees. It is now generally accepted that traditional lectures are poorly suited to teaching and learning in the 21 ^st^ century, with active learning approaches (such as the “flipped classroom” ( Jensen *et al.*, 2015; Lage *et al.*, 2000)) becoming increasingly popular. The ability to carry out practical-based laboratory classes set the sciences apart from many other disciplines, and it is therefore important that these are fully exploited in order to provide the widest possible diversity of active learning approaches.

The unique requirements of developmental biology (discussed above) make this practical approach all the more important. In ‘Practical Developmental Biology’, students were not only given hands-on experience of several key laboratory model organisms (zebrafish, chickens, fruit flies among others), they were also given the opportunity to observe embryonic development for themselves through the regular monitoring of externally-developing zebrafish embryos and the “windowing” of fertilised chicken eggs. Lectures, workshops, textbooks and even videos are no substitute for such experiences, and the obvious fascination of students confronted for the first time by a tiny, beating embryonic heart or developing limb and the eagerness with which they reach for their smartphones to record the event speaks for itself. More basically, the small class size (30) enabled students to work both individually and in pairs and the overlapping nature of the practicals enhanced time-management and self-organisation and this latter was enhanced via the keeping of a combined lab book and learning journal. The requirement to collect, stage and fix embryos of several different species enhanced microscopy and observational skills, and prompted many discussions regarding the issues with assigning distinct stages to what is essentially a continuous process. The use of antibodies to “mystery proteins” and DIG-labelled antisense riboprobes to “mystery genes” (amplified using “mystery primers” by the students themselves, from RNA they extracted and converted to cDNA) ensured that even when following protocols, students were unsure of the outcome, removing the predictable results common to many “interminable, repetitive and boring” undergraduate practical classes ( Adams, 2009). Student feedback showed appreciation for “large variety of practical work”; “gaining more skills in the lab, which a general zoology degree seems to lack”; “learning new techniques” and the “…relaxed, informal atmosphere that encouraged individual work and investigation”.

## Conclusions

Whilst the large class sizes on many undergraduate degrees and the associated implications for physical space and resources (equipment, consumables, technical staff) can often be a barrier to practical classes, there are some subjects where the opportunity to experience size, shape, texture, sights and even smells across days or even weeks cannot be replaced. Developmental biology is one such subject and all the innovative alternatives in the world (including audio-visual resources, flipped classrooms and other active learning approaches) cannot substitute for the sheer wonder of observing embryonic development first hand.
