# Supplementary material for: Developmental biology teaching - the importance of a practical approach
Source: F1000Res. 2015 May 26;4:126. [Version 1] doi: 10.12688/f1000research.6559.1 (PMC4482206; doi:10.12688/f1000research.6559.1)
Supplement: Peer review history

# Peer review: Referee response for version 1

**DOI:** 10.5256/f1000research.7043.r8756

**Published:** 2015-06-08

**Authors:** Thomas Butts

**Affiliations:** 1 School of Biological and Chemical Sciences, Queen Mary University of London, London, UK

This is a thought provoking opinion piece on the advantages, indeed the need, for practical teaching for developmental biology. In general, it is a well written piece that makes a strong case, and one that I whole-heartedly endorse. In the background section, I am a little wary of the approach to thinking about practicals whereby such activities have specific independent purposes that can be expressed in a meaningful way. Are Kirschner's three motives mutually exclusive? I would suggest not, and would avoid discussing them as such. The article describes a practical developmental biology course designed and run by the author that sounds very interesting indeed. If anything, a more thorough description of the course (including its assessment components) and its philosophical underpinnings would be useful and interesting. Also useful would be speculation on how such a course could be 'scaled up', if indeed this is envisaged. Overall, this is a interesting piece that outlines an interesting course that would benefit from more thorough elucidation.

I have read this submission. I believe that I have an appropriate level of expertise to confirm that it is of an acceptable scientific standard.

# Peer review: Referee response for version 1

**DOI:** 10.5256/f1000research.7043.r8755

**Published:** 2015-06-01

**Authors:** Anthony Graham

**Affiliations:** 1 MRC Center for Developmental Neurobiology, Kings College London, London, UK

This is an interesting article that makes a solid case for teaching developmental biology via practical classes employing a range of different species and using a number of different technical approaches. It is clear that the students would gain a lot of valuable insights and depth of understanding of developmental biology by this approach. Actually seeing embryos is something that is likely to grab the imagination of most students. I would ask for a bit more information on some aspects. Could we have a clearer idea of the assessments used? What exactly were the three pieces of course work? I would also like more information on how well the course worked. Were there some areas that were more successful than others? Were some aspects of the course more difficult for the students to master than others? What aspects of the course were most engaging for the students?

I have read this submission. I believe that I have an appropriate level of expertise to confirm that it is of an acceptable scientific standard.

# Peer review: Referee response for version 1

**DOI:** 10.5256/f1000research.7043.r8757

**Published:** 2015-05-29

**Authors:** Graham Scott

**Affiliations:** 1 School of Biological, Biomedical, and Environmental Sciences, University of Hull, Hull, UK

This short opinion piece extols the value of a practical approach to learning  and presents an interesting question: how can we ensure that students both learn facts and concepts/principles. The question is posed in the context of developmental biology but it applies equally well to all disciplinary areas. The author develops/explains this central question well (if briefly). He then presents information about his own practice and in particular describes (very briefly) a module that he has developed and delivered (once). He presents a discussion in which elements of the module are described, and finally he concludes that his discipline is one in which practical experience is essential.

Each of these individual parts is interesting but they are not fully developed and I do not feel that they add up to a discussion of the value of a practical approach to developmental biology, nor do they currently address the question that the author poses.

An opinion piece is of course an encapsulation of the opinions of the author and in this case I agree with the opinion expressed. However for a reader to be able to evaluate these opinions and assimilate them with their own experiences it is essential that they are supported by evidence. Unfortunately I don’t think the author of this paper has achieved this. The link between the introductory material and the description of practice is not a fully developed one. In fact it would be possible to remove the description of practice here without really changing the message that is conveyed (ideally of course the case study should enhance the message). I would recommend that the author further develops the explanation and evaluation of the design of the module in the context of both its purpose and outcomes.

Furthermore the evaluation  presented here is very limited.  As a reader I would like to better understand the author’s aims in designing the module? the level to which these aims are achieved? how  their achievement is measured/assessed? I also feel that a stronger inclusion of the student voice (through an enhanced evaluation of student perception and engagement) would strengthen the argument that this model is right in this context.

Finally it is essential that the conclusion be re-written to make better use of the evidence that the author draws directly upon in formulating his opinion.

I have read this submission. I believe that I have an appropriate level of expertise to state that I do not consider it to be of an acceptable scientific standard, for reasons outlined above.

# Response

**Published:** 2015-06-01

**Authors:** John Mulley

**Affiliations:** Bangor University, UK

Thanks for the helpful review - I'll get to work on incorporating these comments and making the required changes.
